# Efficient Biolistic Transformation of Immature Citrus Rootstocks Using Phosphomannose-isomerase Selection

**DOI:** 10.3390/plants8100390

**Published:** 2019-09-30

**Authors:** Hao Wu, Yosvanis Acanda, Michel Canton, Janice Zale

**Affiliations:** 1 Horticulture Sciences Department, Citrus Research & Education Center, University of Florida, Lake Alfred, FL 33850, USA; 2 Department of Plant Pathology, Citrus Research & Education Center, University of Florida, Lake Alfred, FL 33850, USA; yosvani.acanda@ufl.edu (Y.A.); michel.canton@ufl.edu (M.C.)

**Keywords:** citrus, transgenic, biolistic transformation, particle bombardment, phosphomannose isomerase selection

## Abstract

This research utilized the *E. coli man*A gene encoding phosphomannose isomerase (PMI) selection on sucrose/mannose medium to increase transformation efficiencies after biolistic transformation of two immature citrus rootstock cultivars. Plasmid DNA, containing the *man*A gene and the enhanced green fluorescent protein (e*gfp*) reporter gene, was bombarded into epicotyl explants of immature Carrizo citrange and Swingle citrumelo. GFP positive shoots were micro-grafted onto in vitro grown immature Carrizo rootstocks. Nineteen transgenic Carrizo shoots were obtained from ten paired shots, and eight Swingle shoots from five paired shots. The mean transformation efficiency of Carrizo was 1.9 transgenics/paired shot while the transformation efficiency of Swingle was comparable at 1.6 transgenics/paired shot. The transformants were analyzed by PCR for the presence of transgenes. Southern blot analysis of eight representative Carrizo transgenic events and four Swingle transgenic events showed that all transgenics had one to three copies of the *manA* gene. The PMI enzyme activity in the transgenic lines was confirmed using the chlorophenol red assay.

## 1. Introduction

For the 2016–2017 season, citrus production in Florida was drastically reduced due to Huanglongbing (HLB), also known as citrus greening disease, and Hurricane Irma. Total citrus production was 78,130 million boxes (40.8 kilograms/box), which was down from 291,800 million boxes during the 2003–2004 season [1]. Citrus improvement is complicated by nucellar embryony, which makes the production and selection of hybrids difficult, and by the long juvenile phase, which can last 3 to 20 years. The 2018 National Academy of Sciences report recommended the production of transgenics and gene-edited plants to impart disease resistance because it might be faster than traditional breeding [2].

*E. coli* phosphomannose-isomerase (PMI) enzyme activity is encoded by the *man*A gene and it has been used as a selectable marker in the genetic transformation of several crops, such as almond (*Prunus dulcis* ) [3], apple (*Malus domestica* Borkh.) [4], cassava (*Manihot esculenta* Crantz) [5], maize (*Zea mays* L.) [6], rice (*Oryza sativa* L.) [7], and wheat (*Triticum aestivum* L.) [6,8]. Transgenic Golden Rice (*O. sativa* L.) lines with carotenoids were produced using *E. coli* PMI selection, to avoid selection with antibiotics and to conform to regulatory requirements [9]. Hu et al. [10] showed that phosphomannose isomerase genes from *O. sativa* L., *Arabidopsis thaliana* L. Heynh., and *Chlorella variabilis* were also effective as selectable markers in the genetic transformation of rice and these genes are valuable for cisgenic and intragenic research.

Phosphomannose-isomerase selection has been used with *Agrobacterium-*mediated transformation of immature citrus scions and rootstocks [11,12,13]. Boscariol et al. [11] showed that transgenic sweet orange (*Citrus sinensis* L. Osbeck) scions could be produced using PMI, with transformation efficiencies ranging from 3% to 23.8% depending on the cultivar, but their results were similar to other selection systems. However, Ballester et al. [12] demonstrated that immature citrus transformation efficiencies could be increased to 30% for Carrizo citrange (*Citrus sinensis* Osb. x *Poncirus trifoliata* L. Raf.) and 13% for ‘Pineapple’ sweet orange (*C. sinensis* L. Osbeck) from 4.3% efficiency in both genotypes using only GUS screening in kanamycin-free medium. Dutt et al. [13] illustrated that *man*A fused to *gfp* in a bidirectional fusion arrangement decreased the number of non-transformed, escaped shoots during immature Carrizo citrange rootstock transformation after *Agrobacterium-*mediated transformations.

The *man*A gene confers the ability to utilize mannose and intermediate products are fed into the glycolysis pathway. Mannose is first converted to mannose-6-phosphate by hexokinase, and then mannose-6-phosphate is reversibly converted into fructose-6-phosphate, which is an intermediate of the glycolysis pathway [4]. Thus, the use of the *man*A gene in genetic transformation provides a new pathway for carbohydrate metabolism. Non-transgenic tissue of most species usually lacks substantial PMI activity, and without it, mannose is phosphorylated to mannose-6-phosphate, which accumulates in the cell, blocking glycolysis by inhibiting phosphoglucose-isomerase and causing subsequent growth retardation [4]. Callus tissue cultures that cannot metabolize mannose will form fewer shoots in the presence of mannose compared to selection with neomycin phosphotransferase (*npt*II) or herbicide resistance genes. In contrast, selection with *npt*II or herbicide resistance genes, non-transgenic shoots still develop, but they are unable to detoxify the chemicals and die after exposure to it. Both immature and mature citrus transformation with *npt*II are plagued by thousands of non-transgenic, escaped shoots [14,15,16].

Biolistic-mediated transformation of citrus is more desirable than *Agrobacterium*-mediated transformation for commercializing disease-resistant citrus because, in the United States, one step of the regulatory process can be avoided if no plant pest sequences are introduced. Similar to gene editing, all-citrus sequences bombarded into explants might avoid Animal and Plant Health Inspection Service (APHIS) regulatory oversight, although Environmental Protection Agency (EPA) and Food and Drug Administration (FDA) oversight would still be necessary for both gene alteration technologies. Towards this goal, Wu et al. [16] devised a biolistic protocol for immature Carrizo citrange rootstock explants, but the transformation efficiency was low with selection of *npt*II. An efficient biolistic transformation protocol is critically important for Citrus spp. The current research addresses whether PMI and mannose selection might decrease the number of non-transgenic, escaped shoots and increase transformation efficiency after biolistic transformation. This is the first report of PMI selection after biolistic transformation for citrus.

## 2. Results

### 2.1. Preliminary Test of Explant Sensitivity to Mannose and Shoot Regeneration

Seven combinations of sucrose and mannose: 0 g L^−1^ of sucrose + 30 g L^−1^ of mannose, 0.2 g L^−1^ of sucrose + 29.8 g L^−1^ of mannose, 0.5 g L^−1^ of sucrose + 29.5 g L^−1^ of mannose, 2 g L^−1^ of sucrose + 28 g L^−1^ of mannose, 5 g L^−1^ of sucrose + 25 g L^−1^ of mannose, 10 g L^−1^ of sucrose + 20 g L^−1^ of mannose and 30 g L^−1^ of sucrose + 0 g L^−1^ of mannose, were initially tested with Carrizo citrange explants (Table S1). The combination of 0.2 g L^−1^ of sucrose + 29.8 g L^−1^ of mannose was the most stringent in terms of selection and was used for the transformation experiments.

### 2.2. Biolistic Transformation of Carrizo Citrange

Five experiments, comprised of ten paired shots, were carried out with immature Carrizo citrange rootstock explants to determine the efficacy of PMI in the selection of transgenic shoots after biolistic transformation. The rationale for using immature Carrizo citrange was that it produces many shoots, and this is desirable in protocol development. The protocol of Wu et al. [16] was followed for bundling the explants and bombarding each terminal end of the bundle (a paired shot). A mean of 1.9 ± 0.4 transgenic shoots was achieved for the ten paired shots (Table 1). For these bombardments, 2,565 shoots were screened for GFP fluorescence. Nineteen non-chimeric shoots fluoresced. Eleven GFP fluorescing shoots were micro-grafted onto Carrizo citrange rootstock, eight shoots (72.7%) survived the micro-grafting procedure, and all tested positive with endpoint PCR for *gfp* and the *man*A gene. Then they were examined for gene expression and copy number of the transgenes (see Molecular analyses of the transgenics). Chimeras and fluorescing non-organogenic shoots were excluded from this study.

### 2.3. Biolistic Transformation of Swingle Citrumelo

Three experiments, comprised of five paired shots, were carried out to determine the efficacy of PMI in the selection of developing shoots after biolistics of immature Swingle citrumelo rootstock explants (Table 2). There were two paired shots in the first two experiments, but only one paired shot for the third experiment due to lack of seed availability. A mean of 1.6 ± 0.5 transgenic shoots was produced over five paired shots (Table 2). Eight shoots were positive for GFP fluorescence, six were micro-grafted onto Carrizo citrange rootstock and four survived micro-grafting (66.7%). These four plants were all PCR positive for both *manA* and e*gfp* genes and were used in subsequent molecular analyses.

### 2.4. Molecular Analyses of the Transgenics

**Figure 1:**
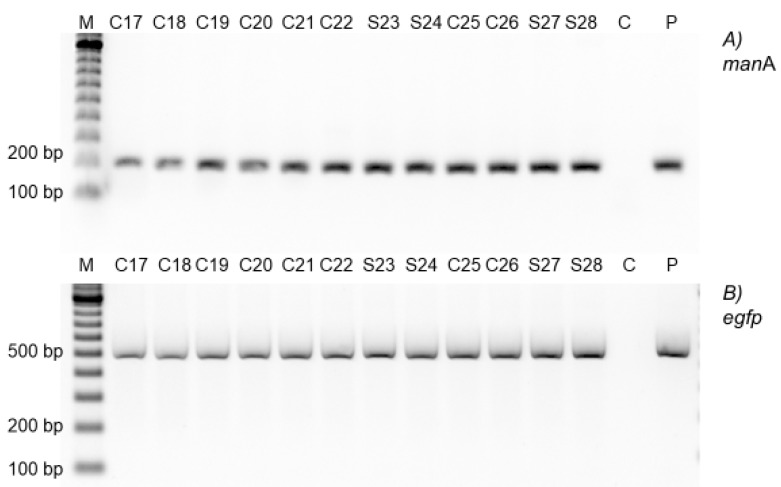
Endpoint polymerase chain reaction (PCR) for *man*A and *egfp* coding regions. (**A**) PCR for the *man*A coding region. (**B**) PCR for the *egfp* coding region. *C*: *Citrus sinensis* Osb. x *Poncirus trifoliata* L. Raf. (Carrizo citrange), *S*: *Citrus paradisi* Macf. Duncan grapefruit x *Poncirus trifoliata* L. Raf (Swingle citrumelo), *M*: Marker is EZ Load 100 bp Molecular Ruler, *P*: p2820eGFP plasmid. Note that only Carrizo citrange was included as a negative control because the genomic background is similar in the two and they do not have *E. coli manA* gene.

Endpoint PCR was used to test for the presence of the *man*A and *egfp* genes, and all of the analyzed Carrizo citrange and Swingle citrumelo transgenic shoots possessed both genes of the correct sizes (Figure 1).

**Figure 2:**
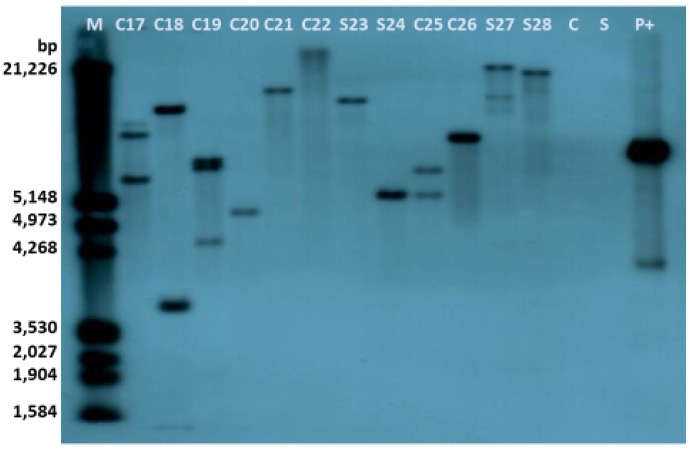
Southern blot analysis using a probe of the *man*A coding region: *C17-22, C25-26*: *Citrus sinensis* Osb. x *Poncirus trifoliata* L. Raf. (‘Carrizo’ citrange) transgenic lines digested with *Eco*RI, *S23-24, S27-28 Citrus paradisi* Macf. Duncan grapefruit x *Poncirus trifoliata* L. Raf (‘Swingle’ citrumelo) transgenic lines digested with *Eco*RI, *C*: ‘Carrizo’ wild type digested with *Eco*RI; S: ‘Swingle’ wild type digested with *Eco*RI, *M*: Molecular marker, *P+*: p2820eGFP plasmid digested with *Eco*RI showing linear and supercoiled conformation due to incomplete digestion.

**Figure 3:**
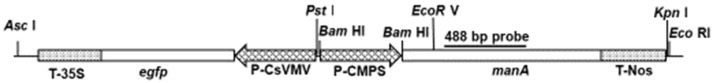
The expression cassettes of plasmid p2820eGFP showing P-CsVMV, the Cassava Vein Mosaic Virus (CsVMV) promoter, e*gfp*, enhanced green fluorescence protein gene, T-35S, the cauliflower mosaic virus 35S terminator, P-CMPS, the Cestrium yellow leaf curling virus promoter, *manA*, mannose-6-phosphate isomerase gene from *E. coli*, T-Nos, the nopaline synthase gene terminator. The size of the expression cassettes is 4.1 kb and the entire plasmid size is 6.4 kb. The probe for Southern blot is 488 bp in *manA* coding region.

Southern Blot analysis of all the available transgenic rootstock events: C17, C18, C19, C20, C21, C22, C25, C26, S23, S24, S27, and S28 showed that all transgenic lines possessed the *manA* gene, and the transgenic copy numbers ranged from one and three copies (Figure 2). The probe description for hybridization is described in Figure 3.

### 2.5. Chlorophenol Red Assay

**Figure 4:**
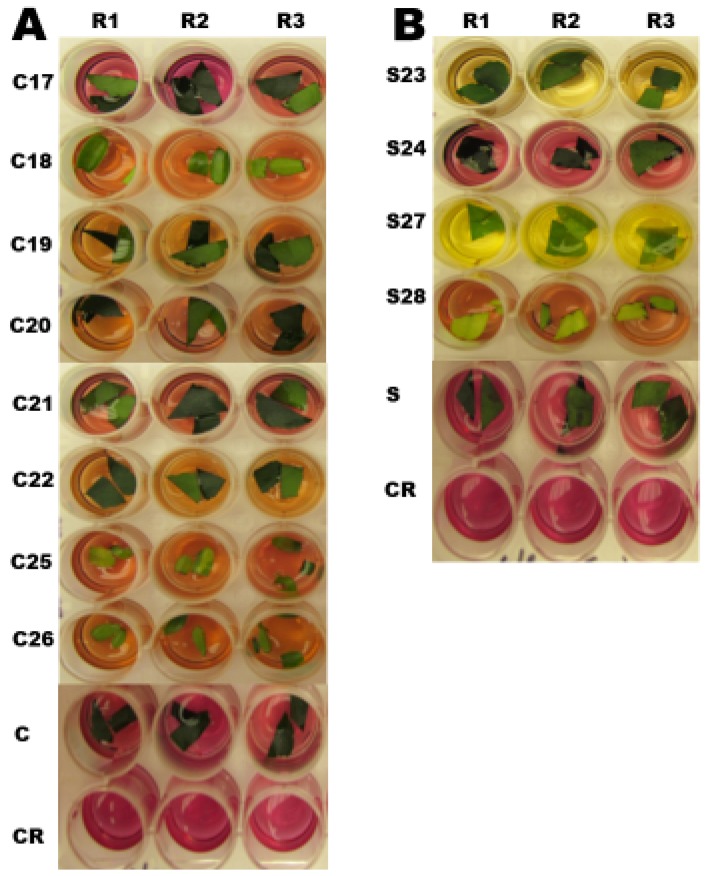
The chlorophenol red pH indicator test for phosphomannose isomerase (PMI). Transgenic, wild-type, and solution alone samples were tested with Murashige and Skoog (1962) plus mannose medium and chlorophenol red pH indicator. A red or purple color indicates negative phosphomannose isomerase activity and an orange or yellow color indicates positive PMI activity. (**A**) Replicated Carrizo samples, Rows *C17, C18, C19, C20, C21, C22, C25, C26*: Leaves of *Citrus sinensis* Osb. x *Poncirus trifoliata* L. Raf. (‘Carrizo’ citrange) transgenics. Row C: Leaves of wild-type ‘Carrizo’, Row *CR*: Chlorophenol red solution alone. (**B**) Replicated Swingle samples, Rows *S23, S24, S27, S28*: Leaves of *Citrus paradisi* Macf. Duncan grapefruit x *Poncirus trifoliata* L. Raf (‘Swingle’ citrumelo) transgenics. Row *S*: Leaves of Wild-type ‘Swingle’, Row *CR*: Chlorophenol red solution alone, R1–R3: Three replicates.

Leaf segments of PCR positive transgenic plants were placed in separate wells of a multi-well plate containing MS liquid medium supplemented with 15 g L^−1^ mannose and 50 mg L^−1^ chlorophenol red, pH 6.0, to confirm PMI enzyme activity in the tissue. A red or purple color indicated no enzyme activity, whereas a yellow or orange color indicated PMI activity and that the tissues were able to utilize the mannose substrate and acidify the medium, changing its color. Visual evaluation was carried out after 5 days of incubation (Figure 4). From the chlorophenol red test, S23 and S27 appeared to express the greatest amount of PMI enzyme. Most of the other samples, except S24, showed some enzyme activity after a 5-day incubation, confirming the transgenic nature of the rootstocks.

## 3. Discussion

In Citrus spp, PMI/mannose selection, using a range of mannose and sucrose concentrations, has been utilized after *Agrobacterium* transformation. An increase in the number of escapes and a reduction in the number of transgenics occurred when the mannose concentrations were lowered or combined with sucrose in the selection medium [11]. Ballester et al. [12] produced a few Carrizo citrange and Pineapple sweet orange transgenics under high selection pressure (0 g L^−1^ sucrose + 15 g L^−1^ mannose). Boscariol et al. [11] utilized 0 g L^−1^ sucrose + 13 g L^−1^ (73 mM) mannose for selection of tansgenics in Pera and Valencia, 0 g L^−1^ sucrose + 15 g L^−1^ (84.2 mM) mannose in Natal, 0 g L^−1^ sucrose + 20 g L^−1^ (112.3 mM) mannose in Hamlin sweet orange. In the present study, high concentrations of mannose (29.8 g L^−1^) combined with low concentrations of sucrose (0.2 g L^−1^) efficiently selected transgenics from multiple experiments of two immature citrus rootstocks after particle bombardment.

Although not directly comparable, the transformation efficiency reported here for Carrizo citrange was 1.9 transgenic shoots per paired shot, which was considerably higher than 0.3 transgenic shoots per paired shot reported by Wu et al. [16]. *Agrobacterium*-mediated transformation is currently the predominant method in Citrus spp. and the efficiency for Carrizo citrange has been reported to be much higher at 44% [17]. To date, there are no commercial transgenic Citrus cultivars in the market possibly due to the time-consuming and expensive regulatory procedures that must be undertaken to develop a transgenic for the market. Biolistic transformation provides a faster track for commercialization because it can bypass the United States Department of Agriculture (USDA) APHIS regulatory oversight step if no plant pest sequences and all plant sequences are used. Since the goal is potential commercialization for the citrus industry, this increase in transformation efficiency after biolistic transformation represents a significant improvement.

When kanamycin was used, the non-transformed cells were killed by the kanamycin, leaving the transgenic cells encapsulated in necrotic tissue [18], and hence, significantly reducing the number of transformed shoots, especially during the early stage of selection. On the other hand, in PMI/mannose selection, non-transformed cells are starved, but not killed, which does not prevent the neighboring transgenic cells from regenerating, and this results in more transgenic shoots than NPTII/kanamycin selection.

The copy number of the transgenes was one to three, which means that in the future, there is a possibility of obtaining single-copy transgenic lines with high expression levels by using less plasmid DNA or bombarding linear, minimal expression cassettes. Wu et al. [19] showed that for sugarcane low copy numbers could be achieved similar to *Agrobacterium*-mediated transformation if linear, minimal cassettes were bombarded. Similarly, in sugarcane, Jackson et al. [20] showed that when the concentration of minimal cassettes was reduced to 6.6 ng per shot, the copy number was similar to *Agrobacterium*-mediated transformation.

*E. coli npt*II confers resistance to the aminoglycoside antibiotics kanamycin and neomycin, and it is probably the most common selectable marker in plant transformation, including citrus. Although *npt*II has been proven safe to humans and the environment [21], there are still concerns about the possibility of horizontal gene transfer to other organisms and increased antibiotic resistance in humans, particularly in the European Union. To allay these fears, different selectable markers, such as *E. co*li PMI, were developed.

Most plant species cannot utilize mannose and have been classified as mannose sensitive [22]. It was suggested that selection with *E. coli* PMI is effective in the transformation of sensitive plant species, such as *A. thaliana* (L.), barley (*Hordeum vulgare* L.), bent-grass (*Agrostis stolonifera* L.), cassava (*M. esculenta* Crantz), cucumber (*Cucumis sativus* L.), flax (*Linum usitatissimum* L.), maize (*Z. mays* L.), onion (*Allium cepa* L.), pearl millet (*Pennisetum glaucum* L.), rice (O. sativa L.), tomato (*Solanum lycopersicum* L.), sorghum (*Sorghum bicolor* L.), sugarbeet (*Beta vulgaris* L.), sugarcane (*Saccharum officinarum* L.), and wheat (*Triticum aestivum* L.), because endogenous PMI enzyme activity might be low [22]. Citrus also has negligible PMI enzyme activity and could be classified into this sensitive category because *E. coli* PMI was effective as a selectable marker and it increases transformation efficiencies. In contrast, mannose-tolerant species, such as salt-tolerant celery (*Apium graveolens* L.) or tobacco (*Nicotiana tabacum* L.), contain significant PMI or the mannose-6-phosphate reductase (M6PR) enzyme activity which metabolizes mannose [22].

Genomic research has revealed the prevalence of PMI sequences in *O. sativa*, *Brassica rapa* and *A. thaliana* [10,23]. The rice PMI homolog functioned well in vitro as a selectable marker in the genetic transformation of rice to provide a cis/intragenic genetic engineering solution [10]. Citrus also has Type I homologs. Future experiments in immature and mature citrus will test the citrus PMI homologs as a selectable marker in genetic transformation experiments to provide an alternative to *E. coli* PMI selection. The consumer might be more willing to accept cis/intragenics rather than transgenic citrus.

## 4. Materials and Methods

### 4.1. Plasmid DNA

The plasmid p2820eGFP was constructed by insertion of the enhanced *gfp* (*egfp*) cassette (Cassava Vein Mosaic Virus promoter::*egfp*::CaMV 35S terminator) from pUCSV-eGFP, kindly provided by Dr. Manjul Dutt at the University of Florida, Citrus Research and Education Center [24,25] into pNOV2820, which carries the *E. coli man*A gene cassette (Cestrium yellow leaf curling virus (CMPS) promoter::*man*A::NOS terminator), obtained from Syngenta (Wilmington, DE) (Figure 3) [26]. To generate p2820eGFP, the plasmid pNOV2820 was linearized with *Hind*III, the ends blunted with Klenow Fragment, and then cut with *Pst*I. Then, it was ligated to the *egfp* reporter gene cassette that was cut with *Eco*RI, then blunted with Klenow Fragment, and cut with *Pst*I. For bombardment, p2820eGFP was prepared from an overnight culture of 100 mL LB *E. coli* strain DH5α at 37 °C using a Plasmid Midi Kit (Qiagen^®^, Germantown, MD, USA).

### 4.2. Plant Materials and Tissue Culture

Seeds of *Citrus sinensis* Osb. x *Poncirus trifoliata* L. Raf. (Carrizo citrange) and (*Citrus paradisi* Macf. ‘Duncan’ grapefruit x *Poncirus trifoliata* L. Raf. (Swingle citrumelo) rootstock cultivars were obtained from Lyn Citrus Nursery in Arvin, CA. The seeds were peeled and sterilized according to the protocol described in Wu et al. [16]. The seeds were germinated and grown on MS medium [27], pH was adjusted to 5.8 with 1 M KOH, plus 2.6 g L^−1^ Phytagel™ (Sigma-Aldrich, St. Louis, MO, USA) for four to five weeks in the dark and then moved to low light intensity of 30 μE^−1^ m^2^ s^−1^ using 17 Watt Philips fluorescence light (F17T8/TL841) (Philips, Somerset, NJ, USA) with a 16 h photoperiod at 26 ± 1 °C (Percival Scientific, Perry, IA) for four to five days. Epicotyls were cut into 1 cm segments and plated onto Murashige and Skoog (MSB) medium supplemented with 5 mg L^−1^ 6-benzylaminopurine (BAP), 30 g L^−1^ sucrose and 2.6 g L^−1^ Phytagel™ four days prior to bombardment or determination of the optimal mannose plus sucrose concentration for selection.

### 4.3. Preparation of Gold Particles

The protocol of Altpeter et al. [28] was followed for one preparation (10 shots) of coating for gold particles (1.0 µm; Crescent Chemical Company, (Islandia, NY, USA) by using 300 ng of p2820eGFP DNA diluted to final ethanol of 100 µL. Five µL aliquots, approximately 15 ng of plasmid DNA, were spread onto each macrocarrier (Bio-Rad^®^ Laboratories, Hercules, CA, USA) for each shot.

### 4.4. Particle Bombardments and Selection on Mannose

Particle bombardment was performed following the protocol outlined by Wu et al. [16]. Briefly, prior to biolistics, the explants were transferred to a high osmotic pretreatment of MS medium with 0.4 M sorbitol for 4 h. Afterwards, the explants were bundled together and both terminal ends (a paired shot) were bombarded in the center of an empty Petri dish using the Bio-Rad^®^ PDS 100/He^TM^ with 10,686.87 kPa Bio-Rad^®^ rupture disks, 6 cm stage height, and a chamber vacuum of 698.5 mm Hg. After bombardment, the explants were released from the ring and transferred directly to MSB medium supplemented with 0.2 g L^−1^ sucrose + 29.8 g L^−1^ mannose (MSB selection medium).

Explant cultures were placed under a low light intensity of 30 μE^−1^m^−2^s^−1^ with a 16 h photoperiod for two weeks at 26 ± 1 °C. Afterwards, the explants were transferred to a higher light intensity of 150 μE^−1^m^−2^s^−1^ with the same photoperiod at 26 ± 1°C, and sub-cultured onto the same fresh selection media every two weeks until shoots developed.

### 4.5. Fluorescence Microscopy and Data Collection

Starting four weeks after bombardment, developing shoots were screened for GFP fluorescence in transformed tissues. Fluorescence was detected with a Nikon SMZ 745T stereoscope (Nikon, Melville, NY, USA). It was equipped with a NIGHTSEA fluorescence adapter (NIGHTSEA, Lexington, MA, USA) and a blue filter, which allows transmission of green light from the GFP protein in transgenic cells. The number of transgenic and non-transgenic shoots was scored eight weeks after bombardment and used in descriptive statistics (Minitab 18).

### 4.6. Micro-Grafting

For micro-grafting transgenic shoots, peeled Carrizo citrange seeds were plated onto sterile seed medium in culture tubes and grown in the dark for four to six weeks [15,29,30]. Some, but not all, GFP positive shoots were micro-grafted onto in vitro grown immature, decapitated Carrizo citrange rootstock seedlings following the protocol described by Wu et al. [15]. At least one micro-graft was performed for each bombardment based on rootstock availability. After the shoots healed onto the immature Carrizo citrange rootstocks, secondary grafts were performed onto six-months-old, soil-grown rootstocks in a growth facility set to a temperature of 28 ± 4 °C, 60% relative humidity and a light intensity of 50–60 μE^−1^ m^2^ s^−1^ using 54 watt GE fluorescence lights (F54W/T5/865/ECO) (General Electric, Boston, MA, USA) with a 12 h photoperiod [15].

### 4.7. Molecular Analyses

Green florescent protein expressing shoots micro-grafted onto immature, wild-type Carrizo citrange seedlings were analyzed by endpoint polymerase chain reaction (PCR) to confirm the presence of the transgenes. Total DNA was isolated from 80 mg fresh, transgenic leaves using DNeasy Plant Mini Kit (Qiagen^®^, Germantown, MD, USA). The primer pairs e*gfp*515 (Fw: CCTGAAGTTCATCTGCACCA; Re: GCTTCTCGTTGGGGTCTTT) and *man*A180 (Fw: ACAGCCACTCTCCATTCAGGTTC; Re: CTCCGGCTTGTGGTTAGGATCT) were used to amplify a 515 bp and a 180 bp PCR product from e*gfp* and *man*A coding regions, respectively. Reactions were set to 50 µL volume containing 0.5 mM of each primer, 0.2 mM of each dNTP, 5 µL of 10X Dream Taq™ Buffer (Thermo Fisher Scientific^®^, Grand Island, NY, USA), 1.25 U of Dream Taq™ DNA polymerase (Thermo Fisher Scientific^®^, Grand Island, NY, USA) and 60 ng of DNA template. The cycling program was run on a MJ Mini™ thermal cycler (Bio-Rad^®^, Hercules, CA, USA) and consisted of a preliminary denaturation step at 95 °C for 3 min, 35 cycles of a denaturation step at 94 °C for 10 s, annealing step at 62 °C for 15 s, and an extension step at 72 °C for 25 s, followed by a 2 min final extension at 72 °C. Polymerase chain reaction products were analyzed by 2% agarose gel electrophoresis with EZ Load 100 bp Molecular Ruler (Bio-Rad^®^, Hercules, CA, USA).

A Southern blot was carried out to determine the transgene copy numbers. Briefly, the cetyl trimethylammonium bromide (CTAB) protocol was used for genomic DNA extraction from young leaves [31]. DNA was digested with *Eco*RI enzyme, which cuts once within the plasmid p2820eGFP but outside of the probe region (Figure 3). The digested DNA was size fractionated in 0.8% agarose gel electrophoresis, depurinated, denatured, and blotted onto Hybond N+ (Roche, Indianapolis, IN) membrane according to the commercial instructions, and the membrane was UV cross-linked at optimum for 2 min. The 488 bp *manA* probe was digoxigenin (DIG) (Roche) labeled by PCR using primers *manA*F2, 5′-ATTATGCGCAGCACAGCCAC -3′ and *manA*R2, 5′-ACAGGAACATCG CTTCGCC -3′ with one cycle of 2 min at 95 °C, 30 cycles of 30 s 95 °C, 30 s 60 °C, 40 s 72 °C, and a final extension of 10 min 72 °C. The DIG labeled probe was evaluated by electrophoresis, and hybridizations carried out according to the manufacturer’s instructions. The blot was exposed to Kodak X-ray film for 30 min prior to image analyses.

### 4.8. Chlorophenol Red Assay

The chlorophenol red assay of [32] was performed to demonstrate PMI enzyme activity in transgenic tissues. The assay was used to test whether transgenic vs. wild-type leaf tissue, approximately 0.5 cm diameter, would metabolize 15 g L^−1^mannose in MS liquid medium with 50 mg L^−1^ chlorophenol red pH indicator, pH 6.0, in microtiter plates. The microtiter plate with tissue pieces was incubated in the dark at 26 ± 1 °C for 5 days and then assessed for color changes.

## 5. Conclusions

The use of PMI selection in the production of immature citrus transgenics after biolistic transformation increases efficiency and decreases non-transgenic, escaped shoots. PMI selection after biolistic transformation generated almost two transgenic events per paired shot, which is efficient for generating a large number of transgenic lines in a short period of time.

## Figures and Tables

**Table 1:** Biolistic transformation of *Citrus sinensis* Osb. x *Poncirus trifoliata* L. Raf. (Carrizo citrange), the number of transgenic shoots produced, micro-grafted, and tested with PCR.

Experiment	Paired Shots	Number of Explants	Number of Shoots	Number of Positive Shoots	Number of Shoots Survived/Number Micro-grafted	PCR Positive Shoots
1	1	153	294	2	0/1	0
1	2	126	237	3	1/2	1
2	3	165	288	4	2/2	2
2	4	153	284	2	1/1	1
3	5	159	270	1	1/1	1
3	6	166	214	1	0/1	0
4	7	154	346	1	1/1	1
4	8	80	140	0	0/0	0
5	9	149	319	3	1/1	1
5	10	88	173	2	1/1	1
Total	-	1393	2565	19	8/11	8/8
Mean ± SE	-	139.3 ± 9.9	256.5 ± 20.5	1.9 ± 0.4	72.7% survival	100%

**Table 2:** Biolistic transformation of *Citrus paradisi* Macf. Duncan grapefruit x *Poncirus trifoliata* L. Raf (Swingle citrumelo) and the number of transgenic shoots produced, micro-grafted and tested with PCR.

Experiment	Paired Shots	Number of Explants	Number of Shoots	Number of Positive Shoots	Number of Shoots Survived/Number Micro-grafted	PCR Positive Shoots
1	1	103	121	0	0/0	0
1	2	99	187	2	2/2	2
2	3	97	132	1	1/1	1
2	4	110	164	2	0/1	0
3	5	144	205	3	1/2	1
Total	-	553	809	8	4/6	4/4
Mean ± SE	-	110.6 ± 8.6	161.8 ± 15.9	1.6 ± 0.5	66.7%	100%

## Supplementary Materials

The following are available online at https://www.mdpi.com/2223-7747/8/10/390/s1; Table S1. Wild-type *Citrus sinensis* Osb. x *Poncirus trifoliata* L. Raf. (Carrizo citrange) explants were tested on seven combinations of sucrose and mannose and shoot regeneration recorded. Fifteen explants were plated onto each plate and each treatment was replicated three times. Shoots that regenerated from the explants were recorded eight weeks after transfer onto medium with the different combinations of sucrose and mannose. The number of shoots that regenerated was used in descriptive statistics, ANOVA for a randomized complete block design, and multiple comparisons using Minitab 18.

## References

[1] USDA, NAS (2018). Florida Citrus Statistics 2016–2017.

[2] National Academy of Sciences, Engineering and Medicine (2018). A Review of the Citrus Greening Research and Development Efforts Supported by the Citrus Research and Development Foundation: Fighting a Ravaging Disease.

[3] Ramesh S.A., Kaiser B.N., Franks T., Collins G., Sedgley M. (2006). Improved methods in *Agrobacterium*–mediated transformation of almond using positive (mannose/pmi) or negative (kanamycin resistance) selection-based protocols. Plant Cell Rep..

[4] Degenhardt J., Poppe A., Montag J., Szankowski I. (2006). The use of the phosphomannose-isomerase/mannose selection system to recover transgenic apple plants. Plant Cell Rep..

[5] Zhang P., Potrykus I., Puonti-Kaerlas J. (2000). Efficient production of transgenic cassava using negative and positive selection. Transgenic Res..

[6] Wright M., Dawson J., Dunder E., Suttie J., Reed J., Kramer C., Chang Y., Novitzky R., Wang H., Artim-Moore L. (2001). Efficient biolistic transformation of maize (*Zea mays* L.) and wheat (*Triticum aestivum* L.) using the phosphomannose isomerase gene, pmi, as the selectable marker. Plant Cell Rep..

[7] Duan Y., Zhai C., Li H., Li J., Mei W., Gui H., Ni D., Song F., Li L., Zhang W. (2012). An efficient and high-throughput protocol for *Agrobacterium*-mediated transformation based on phosphomannose isomerase positive selection in *Japonica* rice (*Oryza sativa* L.). Plant Cell Rep..

[8] Gadaleta A., Giancaspro A., Blechl A., Blanco A. (2006). Phosphomannose isomerase, *pmi*, as a selectable marker gene for durum wheat transformation. J. Cereal Sci..

[9] Hoa T.T.C., Al-Babili S., Schaub P., Potrykus I., Beyer P. (2003). Golden *Indica* and *Japonica* rice lines amenable to deregulation. Plant Physiol..

[10] Hu L., Li H., Qin R., Xu R., Li J., Li L., Wei P., Yang J. (2016). Plant phosphomannose isomerase as a selectable marker for rice transformation. Sci. Rep..

[11] Boscariol R., Almeida W., Derbyshire M., Mourao Filho F., Mendes B. (2003). The use of the PMI/mannose selection system to recover transgenic sweet orange plants (*Citrus sinensis* L. Osbeck). Plant Cell Rep..

[12] Ballester A., Cervera M., Peña L. (2008). Evaluation of selection strategies alternative to *npt*II in genetic transformation of citrus plants. Plant Cell Rep..

[13] Dutt M., Lee D.H., Grosser J.W. (2010). Bifunctional selection–reporter systems for genetic transformation of citrus: Mannose-and kanamycin-based systems. In Vitro Cell. Dev. Biol. Plant.

[14] Febres V., Khalaf A., Moore G.A., Fisher L. (2011). Citrus Transformation: Challenges and Prospects.

[15] Wu H., Acanda Y., Shankar A., Peeples M., Hubbard C., Orbović V., Zale J. (2015). Genetic transformation of commercially important mature citrus scions. Crop Sci..

[16] Wu H., Acanda Y., Jia H., Wang N., Zale J. (2016). Biolistic transformation of Carrizo citrange (*Citrus sinensis* Osb.× *Poncirus trifoliata* L. Raf.). Plant Cell Rep..

[17] Dutt M., Grosser J. (2009). Evaluation of parameters affecting *Agrobacterium*-mediated transformation of citrus. Plant Cell Tissue Organ Cult..

[18] Okkels F., Ward J., Joersbo M. (1997). Synthesis of cytokinin glucuronides for the selection of transgenic plant cells. Phytochemistry.

[19] Wu H., Awan F.S., Vilarinho A., Zeng Q., Kannan B., Phipps T., McCuiston J., Wang W., Caffall K., Altpeter F. (2015). Transgene integration complexity and expression stability following biolistic or Agrobacterium-mediated transformation of sugarcane. In Vitro Cell. Dev. Biol. Plant.

[20] Jackson M.A., Anderson D.J., Birch R.G. (2013). Comparison of *Agrobacterium* and particle bombardment using whole plasmid or minimal cassette for production of high-expressing, low-copy transgenic plants. Transgenic Res..

[21] Breyer D., Kopertekh L., Reheul D. (2014). Alternatives to antibiotic resistance marker genes for in vitro selection of genetically modified plants–scientific developments, current use, operational access and biosafety considerations. Crit. Rev. Plant Sci..

[22] Song G.-Q., Sink K.C., Ma Y., Herlache T., Hancock J.F., Loescher W.H. (2010). A novel mannose-based selection system for plant transformation using celery mannose-6-phosphate reductase gene. Plant Cell Rep..

[23] Wang X., Zhang S., Zhao X., Li Y., Liu T., Wang J., Hou X., Li Y. (2014). *BcPM*I2, isolated from non-heading Chinese cabbage encoding phosphomannose isomerase, improves stress tolerance in transgenic tobacco. Mol. Biol. Rep..

[24] Dutt M., Li Z., Dhekney S., Gray D. (2008). A co-transformation system to produce transgenic grapevines free of marker genes. Plant Sci..

[25] Dutt M., Erpen L., Ananthakrishnan G., Barthe G., Brlansky R., Maiti I., Grosser J. (2016). Comparative expression analysis of five caulimovirus promoters in citrus. Plant Cell Tissue Organ Cult..

[26] Hohn T., Stavolone L., De Haan P.T., Ligon H.T., Kononova M. Cestrum Yellow Leaf Curling Virus Promoters. Google Patents: 2007. https://patents.google.com/patent/WO2001073087A8/en?oq=google+patents+cestrum+yellow.

[27] Murashige T., Skoog F. (1962). A revised medium for rapid growth and bio assays with tobacco tissue cultures. Physiol. Plant..

[28] Altpeter F., Sandhu S., Davey M.R., Anthony P. (2010). Genetic transformation–biolistics. Plant Cell Cult. Essent. Methods.

[29] Cervera M., Juarez J., Navarro L., Pena L., Pena L. (2005). Genetic transformation of mature citrus plants. Transgenic Plants: Methods and Protocols.

[30] Orbović V., Shankar A., Peeples M.E., Hubbard C., Zale J., Wang K. (2015). Citrus transformation using mature tissue explants. Agrobacterium Protocols.

[31] Porebski S., Bailey L.G., Baum B.R. (1997). Modification of a CTAB DNA extraction protocol for plants containing high polysaccharide and polyphenol components. Plant Mol. Biol. Rep..

[32] Kramer C., DiMaio J., Carswell G.K., Shillito R.D. (1993). Selection of transformed protoplast-derived Zea mays colonies with phosphinothricin and a novel assay using the pH indicator chlorophenol red. Planta.

