# Supplementary material for: Efficient Biolistic Transformation of Immature Citrus Rootstocks Using Phosphomannose-isomerase Selection
Source: Plants (Basel). 2019 Sep 30;8(10):390. doi: 10.3390/plants8100390 (PMC6843792; doi:10.3390/plants8100390)
Supplement: Supplementary file 1 [file plants-08-00390-s001.pdf]

Supplementary materials

Efficient biolistic transformation of immature citrus rootstocks using phosphomannose-isomerase selection

Table S1. Wild-type *Citrus sinensis* Osb. x *Poncirus trifoliata* L. Raf. (Carrizo citrange) explants were tested on seven combinations of sucrose and mannose and shoot regeneration recorded. Fifteen explants were plated onto each plate and each treatment was replicated three times. Shoots that regenerated from the explants were recorded 8 wk after transfer onto medium with the different combinations of sucrose and mannose. The number of shoots that regenerated was used in descriptive statistics, ANOVA for a randomized complete block design, and multiple comparisons using Minitab 18.

		**Number of Explants Cultured**				**Number of Shoots Regenerated**			
**Sucrose ($g\ L^{-1}$)**	**Mannose ($g\ L^{-1}$)**	**Plate 1**	**Plate 2**	**Plate 3**	**Sum**	**Plate 1**	**Plate 2**	**Plate 3**	**Means***
30	0	15	15	15	45	97	102	87	95.3 a
10	20	15	15	15	45	83	90	100	91.0 a
5	25	15	15	15	45	89	67	92	82.7 ab
2	28	15	15	15	45	81	78	66	75.0 bc
0.5	29.5	15	15	15	45	60	56	71	62.3 c
0.2	29.8	15	15	15	45	31	24	29	28.0 d
0	30	15	15	15	45	0	0	0	0 e
Total	-	105	105	105	315	441	417	445	-

*Multiple comparisons performed using Fisher's pairwise comparisons. Means that do not share a common value are significantly different at the 95% level.
